# Extensive chronic xanthogranulomatous intra-abdominal inflammation due to *Mycoplasma hominis* mimicking a malignancy: a case report

**DOI:** 10.4076/1752-1947-3-9211

**Published:** 2009-09-11

**Authors:** Luc Biedermann, Dominik J Schaer, Matteo Montani, Rudolf Speich, Beat Müllhaupt

**Affiliations:** 1 Department of Internal Medicine, University Hospital Zurich, Raemistrasse 100, CH-8091 Zurich, Switzerland; 2 Department of Clinical Pathology, University Hospital Zurich, Schmelzbergstrasse 12, CH-8091 Zurich, Switzerland; 3 Swiss HPB (Hepato-Pancreato-Biliary) Center and Department of Gastroenterology and Hepatology, University Hospital Zurich, Raemistrasse 100, CH-8091 Zurich, Switzerland

## Abstract

**Introduction:**

While infectious peritonitis is a common occurrence in patients with liver cirrhosis, *Mycoplasma* is rarely identified as a causative agent.

**Case presentation:**

We report the case of a 43-year-old Caucasian woman presenting with an extensive abdominal conglomerate tumor mimicking malignancy. A histologic specimen showed a xanthogranulomatous inflammation. Subsequently, *Mycoplasma hominis* was identified as the specific causative infectious agent using a broad-range (eubacterial) polymerase chain reaction. To the best of our knowledge, this is the first reported case of an intra-abdominal *Mycoplasma* infection presenting as a conglomerate tumor.

**Conclusion:**

An unusual presentation of an inflammatory process in the abdomen or an insufficient response to conventional therapy should prompt clinicians to consider atypical infectious agents in the differential diagnosis. This case illustrates the potential of newer diagnostic methods, since certain fastidious microorganisms may not be diagnosed and treated appropriately using conventional means.

## Introduction

The term pelvic inflammatory disease (PID) refers to the inflammation of the upper female genital tract (endometrium, fallopian tubes and contiguous structures) following an ascending infection. It is the most frequent cause of the need for emergency treatment in gynecological departments [1].

The most important sequelae of PID in women of childbearing age are infertility, ectopic pregnancy and chronic pelvic pain. It is often difficult to determine the disease's causative agent because, apart from the usual pathogens such as *Chlamydia trachomatis* and *Neisseria gonorrhoeae*, uncommon agents such as *Mycoplasma hominis* have to be considered as well [2,3]. The role of *Mycoplasma hominis* as a causative pathogen in PID has been demonstrated in a pelvic specimen obtained through laparoscopy [4].

Xanthogranulomatous inflammation is characterized by granulation tissue, foamy histiocytes and sporadic multinucleated giant cells without the occurrence of true granuloma formation. It has also been reported in the gall bladder, kidneys and female genital tract [5].

We report the case of a patient with a partly necrotizing, severe and impressively extensive intra-abdominal inflammation that mimicked a malignancy. The inflammation could be attributed to a smoldering infection with *M. hominis*, most likely a sequel of a tubo-ovarian abscess. Diagnosis was delayed due to a confounding primary diagnosis of chronic hepatitis C (CHC) infection with concomitant hydropic decompensation and renal failure, as well as biologic features of *M. hominis* hindering its detection by conventional means. The key to establishing diagnosis was a biomolecular approach using a broad-range (eubacterial) polymerase chain reaction (PCR) test.

## Case presentation

A 43-year-old Caucasian woman was admitted to a peripheral hospital due to continuous and diffused abdominal pain for one week prior to presentation. A laparoscopic cholecystectomy was performed. Intraoperatively, the surgeon noticed an irregular surface on the liver consistent with liver cirrhosis, although unfortunately no liver biopsy was obtained. The surgical procedure and the postoperative course were uneventful. CHC infection was diagnosed serologically as the cause of the asymptomatic liver disease. Tests for other infectious agents including HIV, Epstein-Barr virus, cytomegalovirus, brucellosis and leptospirosis, as well as for metabolic liver disease were negative. A week after cholecystectomy, the patient was transferred to the intensive care unit because of continuing diffuse abdominal pain, progressive oliguria, hypotonia and anasarca. Ascites was detected by ultrasound. Paracentesis revealed a markedly elevated white blood cell count (27600/µl, 90% neutrophils) and low serum-ascites albumin gradient (7 g/dl). *Streptococcus oralis* (sensitive to all tested antibiotics) grew in the ascites culture but not in the blood cultures. Treatment with intravenous ceftriaxone was thus begun. An abdominal computed tomography was performed because of the patient's abdominal pain and slightly elevated amylase levels. Besides the moderate amount of ascites the only pathological finding was an incidental cystic lesion in the patient's right adnexa.

The patient was subsequently admitted to our hospital due to persistent liver dysfunction (Child-Pugh class B (9 points), Model for End-Stage Liver Disease (MELD) score 12), bacterial peritonitis and impaired renal function (minimal estimated glomerular filtration rate (GFR), with the Modification of Diet in Renal Disease (MDRD) formula was 23 ml/min).

Soon after she was transferred to our hospital, the patient exhibited hepatorenal syndrome, which was successfully treated with terlipressin. Repeated paracentesis was necessary to relieve respiratory compromise and abdominal distension. Cytological analysis didn't reveal any evidence of malignant cells. Cell count continuously decreased to 400 cells/µl, with 3% neutrophils but 45% lymphocytes, some of the latter with reactive changes; the first paracentesis in our hospital revealed 4200 cells/µl with 64% neutrophils and 15% lymphocytes. There was no bacterial growth in any of the ascites samples or blood cultures. Given the prolonged critical condition of the patient, elevated white blood cell count, ascites and elevated C-reactive protein level, the patient's antibiotic therapy was switched from ceftriaxone to piperacillin and tazobactam.

**Figure 1:**
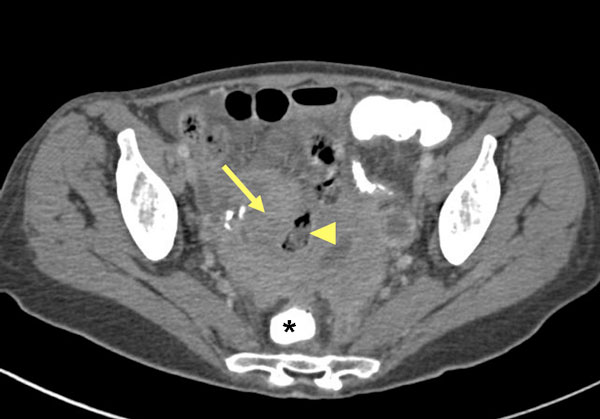
**Computed tomography of the pelvis**. A large conglomerate tumor *(arrow)* with hypodense and isodense fractions that surrounds and partly compresses intestinal loops of the ileum *(arrowhead)* is shown. There is no sharp demarcation of the lesion to the rectum that is filled with contrast agent *(asterisk)*.

**Figure 2:**
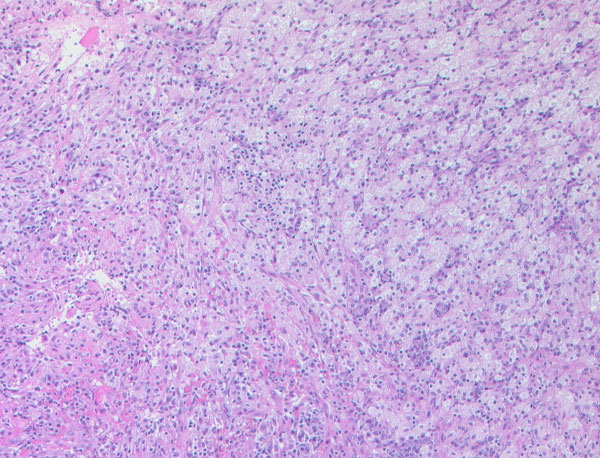
**Biopsy specimen (hematoxylin and eosin) of the removed conglomerate tumor**. A mixed, predominantly mononuclear, cellular inflammatory infiltrate, partially with foamy cytoplasm together with areas with fibrinoid necrosis is shown.

Since both the repeated ultrasound examinations and computed tomography scans (Figure 1) showed a steady increase in the size of the cystic tumor of the adnexa, a diagnostic laparotomy was performed. Strikingly, there was an extensive inflammatory reaction in the patient's lower abdomen with a conglomerate tumor encompassing the uterus and adjacent parts of the sigmoid, rectum and ileocecal junction with crumbly, partly necrotic tissue in between. The whole of the conglomerate tumor was removed. Histology of all examined tissue samples revealed chronic, partly sub-acute inflammatory changes with a xanthogranulomatous pattern and there was no evidence of malignancy (Figure 2).

The patient had no history of mycobacterial infection or whole blood interferon γ assay. PCR analysis for mycobacteria from adnexa tissue and ascites was also negative. However, results of eubacterial PCR of ascites showed positive for *M. hominis*. The same pathogen grew in repeated cultures of ascites, this time using a special culture medium.

## Discussion

This case illustrates the difficulty in identifying the causative agent in a case of infectious peritonitis with an extensive intra-abdominal inflammatory reaction, especially considering the confounding factors present in this case, including cholecystectomy and decompensated liver disease due to chronic hepatitis C infection. However, this case demonstrates the importance of an aggressive approach using laparoscopy or even laparotomy, as well as the value of newer biomolecular culture-independent techniques, in the diagnostic evaluation of such inflammatory reactions.

Infections with *Chlamydia* and *Gonococcus* are well described in the literature representing the most frequent cause of PID and its secondary complications like peritonitis. However, repetitive analyses of cervical smear, urine and ascites using PCR for these two pathogens were always negative in our patient, and thus virtually excluded such a diagnosis. Both gynecological infections are mainly acquired via heterosexual contact in women in their reproductive years, and this explains the high rate of co-existence of both causative agents.

Gynecological infections potentially increase the transmission of and the risk of infection with HIV. On the other hand, HIV infection clearly increases the susceptibility for infectious diseases including PID. There are conflicting data on whether HIV infection influences the spectrum of pathogens responsible for PID or not, but there seems to be a higher incidence of adnexal masses and tubo-ovarian abscesses in women with HIV infection and PID. Furthermore, genitourinary tuberculosis is more common in women with HIV infection and the genitourinary system is reported to be involved in at least a third of all extra-pulmonary cases of tuberculosis. Interestingly, the association between tuberculosis and HIV is considered a substantial public health threat, particularly in developing countries.

In this case, considering the intraoperative findings after diagnostic laparotomy, as well as the patient's history of fever and weight loss, tuberculosis was a main concern.

However, in our patient, PCR for mycobacteria from both the ascites and histologic specimens were negative. Although this method only has a relatively low sensitivity, the negative whole blood interferon γ assay, as well as no personal history of contact with tuberculosis and a normal chest X-ray, strongly argued against a diagnosis of tuberculosis. HIV infection had already been ruled out by serologic testing in the peripheral hospital. Negative test results were also confirmed after admission to our hospital.

Another potential mechanism for the findings in our patient was possible spillage of gallstones into the peritoneal cavity, which is a well-known complication leading to inflammatory changes that may sometimes present as mass lesions [6]. The lack of pigmented material in tissue analyses and the uneventful removal of the gallbladder strongly argued against this cause.

The exact pathogenesis of xanthogranulomatous inflammation remains unclear. Various possible causes such as infection, ineffective antibiotic therapy, irradiation, endometriosis, abnormalities in lipid metabolism and ineffective clearance of bacteria by phagocytes, have been suggested. It has been suggested that after the occurrence of tissue necrosis, cholesterol and other lipids are released and subsequently phagocytosed by macrophages [7]. Knowledge of this entity is important, as it may mimic malignancy. Among others, infectious agents *Escherichia coli*, *Proteus vulgaris*, *Enterococcus spp.* and *Proteus magnus* have been described [8]. However, these micropathogens do not typically play an etiological role in PID.

Phenotypic assessments including morphology and staining behavior, growth factor requirements, fermentation and assimilation of specific carbohydrates are still the mainstay in the identification of infectious agents. Genotypic methods as an alternative approach have gained more popularity for bacterial phylogeny, the identification of non-cultivable pathogens and the differentiation of cultured microorganisms. The small-subunit (16S) ribosomal RNA (rRNA) gene comprises relevant phylogenetic information, and this renders it a suitable gene for amplification and identification [9]. After sequence determination following amplification of 16s ribosomal DNA from species-specific sections, the use of large comparative databases may allow the allocation of the unidentified pathogen to a specific group of bacteria [10].

The use of these culture-independent techniques is especially useful in detecting fastidious microorganisms that are difficult to cultivate and identify [11], as in the analysis of resected heart valves in culture-negative endocarditis [12]. Molecular identification may be superior to standard identification procedures for isolates not revealing a reliable identification result when using standard techniques [11].

*M. hominis* and *Ureaplasma urealyticum* are frequently isolated in the lower urogenital tract in healthy adults, although they are associated with genital infection only in women.

Extragenital infections with *M. hominis*, such as septicaemia, infection of hematoma, prosthetic valve endocarditis and peritonitis, are well known. There are only a few case reports of patients with peritonitis after renal transplantation (n = 6), on chronic ambulatory peritoneal dialysis (n = 1) or after liver transplantation (n = 1) [13]. Thus, most of the recorded cases of extragenital *M. hominis* infection have associated it with immunosuppression [14]. Although speculative, we consider this patient's liver cirrhosis was a contributing factor in the development of this extensive infectious complication. Susceptibility to infection in liver cirrhosis may be due to multiple factors, such as cytokine dysfunction altering the inflammatory response, impaired cellular immunity and hemodynamic dysfunction with systemic vasodilatation [15].

The main structural characteristic of *Mycoplasma* is the lack of cell wall, which makes it innately resistant to β-lactams and to all other antibiotics that target the cell wall. In our patient, the antibiotics administered initially (ceftriaxone and piperacillin/tazobactam) were directed against common causative agents of bacterial peritonitis but did not cover *M. hominis.* This allowed the continued expansion of the infection. Bacterial cultures on appropriate medium that are specifically suitable for growth of *M. hominis*, *M. fermetans* and *U. urealyticum* from the last paracentesis sample were ordered after sequences of 16S-rRNA gene specific for *M. hominis* were detected. The cultures showed growth of *M. hominis* and thus proved a persistent intra-abdominal infection. Antibiotic therapy with doxycycline was initiated with consequent improvement of the clinical condition of the patient and the resolution of inflammatory markers in her blood.

## Conclusions

This case underlines the importance of considering atypical infectious agents in unexplained inflammatory peritoneal processes even if conventional microbiological methods reveal no specific pathogens. Modern biomolecular approaches may be helpful in such instances.

## Abbreviations

CHC: chronic hepatitis C; GFR: glomerular filtration rate; PCR: polymerase chain reaction; PID: pelvic inflammatory disease; MDRD: Modification of Diet in Renal Disease; MELD: Model for Endstage Liver Disease.

## Competing interests

The authors declare that they have no competing interests.

## Consent

Written informed consent was obtained from the patient for publication of this case report and any accompanying images. A copy of the written consent is available for review by the Editor-in-Chief of this journal.

## Authors' contributions

LB managed the patient, conceived the initial idea and drafted the manuscript. DJS and BM analyzed and interpreted the patient data and managed the patient, BM interpreted the data related to hepatic disease. MM performed the histology examination. RS was a major contributor in writing the manuscript and the critical review. All authors read and approved the final manuscript.
